# UK women smokers' experiences of an age-progression smoking cessation intervention: Thematic analysis of accounts

**DOI:** 10.1016/j.pecinn.2022.100021

**Published:** 2022-02-07

**Authors:** Lucy Walker, Sarah Grogan, Keira Scholtens, Andrew Denovan, Brian McMillan, Christopher J. Armitage, Mark Conner, Tracy Epton, Maria I. Cordero

**Affiliations:** a Department of Psychology, Manchester Metropolitan University, Manchester, UK; b Centre for Health Psychology, The Science Centre, Staffordshire University, Stoke on-Trent, UK; c Adelphi Values Ltd, Bollington, UK; d Centre for Primary Care and Health Services Research, University of Manchester, Manchester, UK; e Division of Psychology and Mental Health*,* Manchester Centre for Health Psychology*,* University of Manchester, Manchester University NHS Foundation Trust, Manchester Academic Health Science Centre*,* NIHR Greater Manchester Patient Safety Translational Research Centre*,* Manchester, UK; f School of Psychology, University of Leeds, Leeds, UK; g Division of Psychology and Mental Health*,* Manchester Centre for Health Psychology*,* University of Manchester*,* Manchester, UK

**Keywords:** Smoking, Age-progression, Intervention, Women, Aging

## Abstract

**Objectives:**

Appearance-related interventions to promote healthy behaviour have been found effective to communicate health risks. The current study aimed to explore women smokers' experiences of age-progression software showing the effects of smoking on the face.

**Methods:**

A qualitative design was implemented, utilizing both individual interviews and focus groups within a critical realist framework. Fifteen, 19–52 year-old women smokers were administered an age-progression intervention. All participants responded to the intervention, engaged in semi-structured interviews, and were invited back to attend one of three focus groups. Data were analysed using inductive thematic analysis.

**Results:**

Four main themes were identified: Health versus Appearance, Shock Reaction, Perceived Susceptibility, and Intention to Quit. Participants found the intervention useful, voicing need for a comprehensive approach that includes both appearance and health. Despite increases in appearance-based apps which could diminish impact, women's accounts of shock induced by the aged smoking-morphed images were similar to previous work conducted more than ten years previously.

**Conclusions:**

The study provides novel insights in how women smokers currently perceive, and react to, an age-progression intervention for smoking cessation.

**Innovation:**

Findings emphasise the implementation of this intervention type accompanied by health information in a range of patient settings.

## Highlights

• Experiences of an age-progression intervention were explored in women aged 19–52
• Shock was experienced despite increased availability of facial-ageing technology
• Focus on health should be accompanied with appearance to impact smoking behaviour
• The intervention could be implemented in patient settings for health promotion

## 1 Introduction

Smoking cessation continues to be an international public health priority [1]. Previous research suggests that women smokers have specific challenges associated with smoking cessation due to being more sensitive to the rewarding properties of smoking and increased reports of positive affect from smoking compared to men [2,3], they therefore may benefit from tailored intervention approaches. Furthermore, women have specific health issues associated with smoking such as increased risk of heart disease [4] and increased issues in fertility [5]. It is therefore important that research informs approaches to support women in smoking cessation.

Smoking cessation interventions tend to focus on negative health impacts and, although this is important, alternative appearance-related techniques have also demonstrated success with women smokers [6]. Focus groups with young women suggested that motivation to quit smoking could be increased by showing the effects of smoking on an individual's own face [7]. Academic interest in interventions that focus on appearance has grown in the last decade. Interventions of this type demonstrate the consequences of health behaviours on our physical appearance in an attempt to draw attention to the broader effects of the behaviour and personalize the risk [8]. Interventions have utilized the availability of software that displays realistic effects of smoking [6,9], alcohol [10], and UV exposure [11]. The majority of these software programmes work by displaying a time progression of the ageing process on a photograph of the face, considering the impact of the health behaviour informed by research on skin ageing. Interventions of this type have more recently been developed into apps and implemented within both schools [12,13] and a range of patient centered healthcare settings such as community pharmacies [9] and waiting room settings [14] with success in educating participants and patients as to the outcomes of smoking and changing behaviour.

Quantitative research on age-progression facial morphing interventions has specifically found them to impact significantly on smoking outcomes in women [9,15]. Qualitative work has also investigated the experiences of women smokers aged 18–34 years, identifying common themes such as a surprise or shock reaction and intentions to quit smoking related to perceived personal relevance of the intervention [16]. Western media problematize women's appearance and ageing [8], and clearly it is important to avoid exacerbating this. However, previous work has shown that facial aging interventions can enable women to accept natural ageing while raising awareness of health damaging behaviours with detrimental effects on the skin [17]. This suggests that appearance may be harnessed in a positive way to promote healthy behaviours such as quitting smoking; however, the context in which these perceptions were formed in the previously cited literature is greatly different than today.

The use of, and popularity of apps that highlight changes in facial appearance has risen in the past decade [18], with built in editing features available in most social media platforms such as Instagram and Snapchat [19]. Also, the industry for apps that monitor and promote health is constantly developing [20]. The only previous published qualitative exploration of a smoking age-appearance facial morphing intervention was conducted in a sample of UK women smokers more than ten years ago [16], when women were less likely to have been exposed to facial morphing technology. Therefore, due to increased familiarity with apps that highlight changes in facial appearance, it might be expected that women smokers might react differently to these kinds of current apps.

For the current study, in light of changes in both facial ageing technology and online health monitoring, the present research aimed to explore women's experiences of an age-progression intervention designed to encourage smoking cessation. Specifically, the research aimed to explore participants' reactions to the intervention, and the potential impact of viewing the intervention on smoking. Developments in age-progression interventions continue to grow internationally in patient centered health settings [9,14] and in schools [12,21,22]. It has been over a decade since Grogan et al.'s [16] qualitative investigation into the experiences of women engaging with an age-progression intervention for smoking, and the popularity of face ageing and face changing apps [18] and use of other health technologies has risen. In light of this, a new exploration of the experience of age-progression interventions is timely in order to better inform how the intervention can be best communicated in healthcare settings moving forward.

## 2 Materials and methods

### 2.1 Design

A qualitative approach was adopted combining individual interviews and focus groups with the same participants. The combination of two data collection methodologies enabled enhanced richness of data and enabled confirmation of themes [23]. Interviews enabled in depth exploration for each individual participant [24]. Focus groups allowed for group interaction and discussion of sensitive issues [25] and a reduction in the power differences between interviewer and interviewees observed in interview settings, empowering participants to speak more freely [26]. Conducting both interviews and focus groups also aided in the analysis of the current findings, as major themes identified within interviews were confirmed within focus groups, aiding rigour of data analysis. A critical realist epistemological position was adopted in the analysis [27]. This approach assumes that participants' accounts do not directly reveal the ‘truth’ of their experiences; instead, interpretation is needed to understand fully participants' experiences with complex health behaviours and interventions [28,29]. The authors of this study recognized that accounts from participants could impact understanding of participants' experiences, yet the researchers also had a role in constructing this knowledge. Consequently, reflexive practice (where we took a critical stance towards our role as researchers) [30] was engaged in throughout the research process, to understand the role of the researcher in constructing the knowledge produced.

### 2.2 Participants

A total of 15 participants took part in the study. Participants were eligible if they were women aged 18–55 years, self-identified as a smoker, and smoked at least one cigarette a week. The sample captured social and casual smokers who previously have been found to have a perceived immunity to health-based messages about the impact of smoking [31]. Participants were asked to exclude themselves if they anticipated distress from discussing topics of health and body image. The upper age limit of 55 was set due to the limitations of the APRIL software as the aging process only covers up to 72 years, therefore it was deemed better to set a minimum lapsed time of 15–17 years to enable the software to display visible age morphing effects.

Participants were recruited from [blinded for review] University (*n* = 9) including staff (*n* = 1) and students (*n* = 8), and from the general public (*n* = 5). The sample comprised mainly white women (*n* = 12), with the exception of three participants who identified as Asian. Ages ranged from 19 to 52 years (*M* = 29 years, *SD* = 10.7), with ten participants aged below 34 years and five aged 34 and above. Participants' smoking habits ranged from one to 20 cigarettes a day. University students and staff were recruited through printed and online advertisements, and Psychology students received points for taking part that contributed towards their study. Participants from the general public were recruited via snowballing from existing contacts. All 15 participants took part in individual interviews. Seven of the same participants agreed to take part in one of three focus groups, conducted between one and four weeks after the intervention session, with an average time of two weeks. Initial analysis of the data ensured that themes were fully saturated, indicating no new information or themes were observed in the data [32]. At this point no further participants were recruited.

See Table 1 for information regarding participants pseudonyms, ages and the number of cigarettes usually consumed in a day.

**Table 1:** Participant Information.

Pseudonym	Age in years	Cigarettes a day
Charlotte	22	1–5
Naomi	23	1–5
Grace	22	1–5
Anna	21	1–5
Laura	39	16–20
Zara	43	6–10
Suzy	34	6–10
Ella	22	6–10
Elaine	48	11–15
Claire	52	16–20
Candice	22	1–5
Mary	25	1–5
Simone	24	1–5
Jody	19	11–15
Sarah	20	1–5

### 2.3 The age-progression intervention

The intervention utilized software to demonstrate the ageing effects of smoking on the face. Facial morphing was achieved, whereby two images of an individual were aged incrementally to display how the participant is likely to age up to age 72 years. One image displayed the natural ageing process, the other displayed ageing with the effect of smoking. The women were shown a series of morphing processes, including both 2D and 3D morphed images, as in previous research [15,16,32].

#### 2.3.1 Intervention Software

The APRIL® age-progression software Version 2.7 [35] displays a series of images of a participant's face as it changes with age. By taking a photograph of an individual's face, and considering physical features such as age, ethnicity and gender, the software illustrates how a person is likely to age up to age 72 years. Brightness and contrast filters are applied as necessary. Facial features detection points (e.g., mouth, eyes, etc.) are manually matched between the participant's picture and the stock image. The software displays a time progression of the ageing process on the individual's photograph. On the left-hand side of the screen, the intervention displays ageing without the effects of smoking following the natural ageing process; on the right-hand side the effects of smoking on the ageing process are displayed. The wrinkling effects of smoking are based on average ageing characteristics taken from a database of 3D scans of smokers and were informed by research findings [35].

### 2.4 Materials

A semi-structured interview guide was developed with open-ended questions, based on Grogan et al. (2010). Topics covered attitudes towards the intervention (e.g. ‘what did you think about the intervention?’), and feelings towards the intervention (e.g. ‘how did you feel when you were doing it?’). The same interview guide was used for both interviews and focus group discussions.

A 22-year old woman (first author) non-smoker, conducted all 15 individual interviews and the three focus groups. Participants were not made aware of the interviewer's smoking status.

### 2.5 Procedure

Ethical approval was granted for the research through [blinded for review] University Research Ethics Committee. Data were collected between April and November 2018. The intervention sessions took place in a quiet room at the [blinded for review]. Participants were provided with information regarding the study and given time to ask any questions before consent was taken. After a short rest period, audio recording and the intervention commenced. The intervention software procedure was followed which included initial photo being taken and calibrated, followed by the display of the aged image sequences. The software display lasted on average 15 min, and ranged from 10 to 25 min depending on participant engagement with the open-ended questions. Images of the participant were deleted following the intervention procedure.

Interviews took place directly after the intervention and lasted around 15 min, and participants were asked to sit comfortably to answer questions regarding the intervention. All participants were asked the same questions in a similar order and funnelling techniques [35]; the interview guide included general questions after which prompts and follow up questions were used to enable participants to expand on their initial responses.

Participants were invited back to participate in focus groups, organised after blocks of five participants had been recruited and completed intervention sessions. The duration of the focus groups was around 40 min and were run similarly to individual interviews. The interviewer acted as moderator to allow all participants the opportunity to intervene in the discussion [27]. Focus groups were kept small due to the sensitive and personal nature of the intervention [36]. As participants had varying time since viewing the intervention image, memory of the intervention image effects was refreshed through displaying to the participants an image of the interviewer's face morphed with the software. The visualization technique has previously been found effective in triggering participants responses specifically to complex health behaviours [35,37].

On completion of the study, participants were debriefed fully including given information on how to access quit smoking support via the UK NHS website and given contact details the research team and either their university counselling support or their GP, if they felt any adverse effects from viewing the intervention images.

**Fig. 1:**
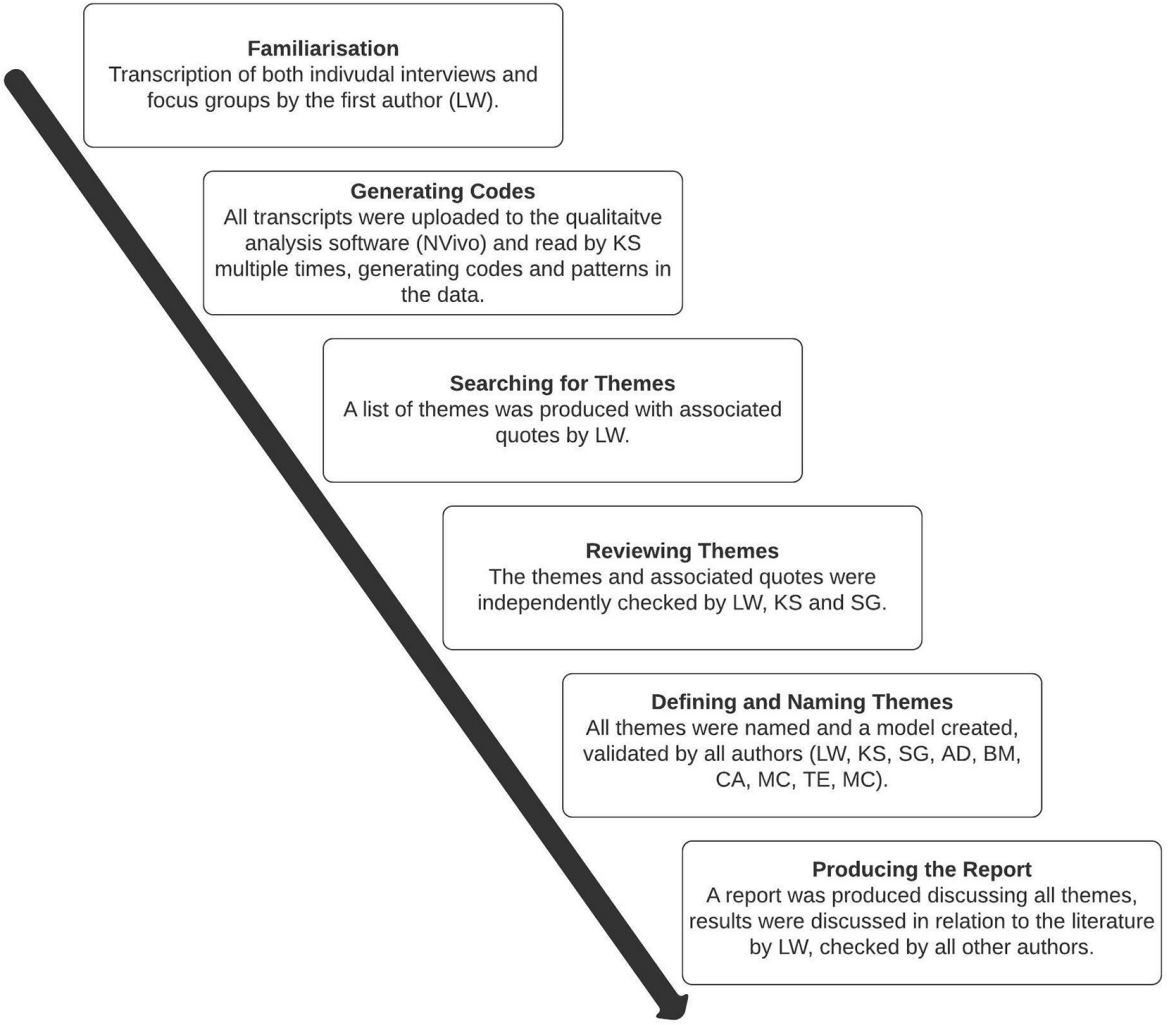
Age-progression intervention Thematic analysis flow chart. Note: Figure displays the steps of thematic analysis taken. Authors initials blinded for review.

All data were transcribed, and analysed using inductive thematic analysis [38], to allow for a detailed interpretation of the participants account of the intervention. The first author transcribed all interview and focus groups recordings. The transcripts were uploaded to qualitative analysis software NVivo 11 (QSR International Pty Ltd) and were read by the first author several times (familiarization process). Coding was conducted line by line in search of themes and patterns within the data whilst making notes. Data from individual interviews were analysed first, followed by that from focus groups to assess whether themes could be confirmed. A list of themes with associated quotes was developed by the first author. The second and third author discussed the thematic structure and quotes at each stage of the analysis process and independently checked the list of quotes to control for bias in interpretation; See Fig. 1 for a diagram of the analysis process.

## 3 Results

**Fig. 2:**
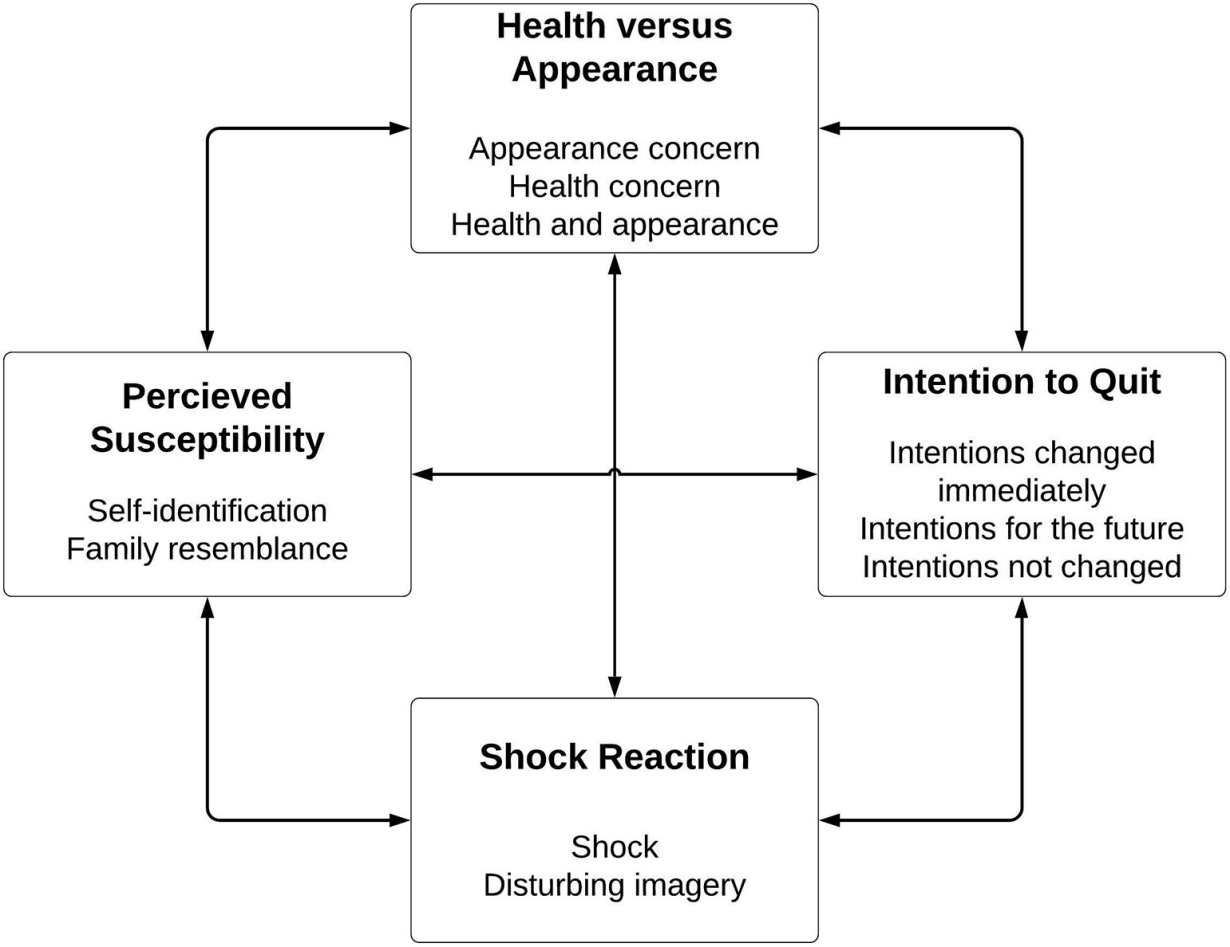
Thematic Map.

Four themes were identified based on data from individual interviews and confirmed through focus group discussion with the same participants. All quotes demonstrating themes are verbatim; only non-audible speech has been removed. Themes and associated quotes are presented in Table 2. Fig. 2 illustrates all themes in bold, and links between themes and sub-themes.

**Table 2:** Example quotes from individual interviews and focus groups.

Theme	Exemplary quote (Pseudonym, age, amount smoked and quote source)
Health versus Appearance	
Appearance Concern	‘Like you get wrinkles where you just generally do when you get old but on the smoking one you are getting them pretty much everywhere on your cheeks and under your eyes are like really black which is one thing I actually do worry about’ [Claire, 52, 16–20, I].
	‘to see it actually like the physical affects because obviously … the internal stuff but you can't see that and I don't know (.) yeah it's just scary comparing it and as bad as it sounds its really scary something could happen to the inside but it's not what you see every day (.) I think that influences me more to be like maybe I should stop because I don't wanna look like that when I'm forty’ [Candice, 22, 1–5, I].
Health Concern	‘the intervention would have worried me more if the visual effects of aging would have been accompanied with like and also you are gonna have trouble breathing or you are gonna have health problems (.) cus yeah I don't really mind how I'm gonna look when I get old’ [Simone, 24,1–5, FG].
	‘I was more worried what it would do inside whether it would slow me down (.) you know what I mean?…but that's what made me think well it does that to your skin what does it do on the inside you know’ [Suzy, 34, 6–10, I].
Health and appearance	‘I've gone to the doctor and asked her about things erm so I have um what you call it inhaler? yeah those ones and then I also have a vape thingy but I don't really like it (.) so I'm taking the steps I'm not saying I'm quite (.) I'm free erm but I have started cutting down so I would say it's all came together with my health and everything and my mum nagging me having to go outside every time so maybe the intervention came at the right time’ [Mary, 25, 1–5, FG].
Shock Reaction	
Shock	‘when you see it it was just a bit like oh shhh (.) can I swear in this? [Interviewer: yes you can if you like] so it's a bit like oh shit (.) like Jesus its quite a (.) I'm gonna say hard hitting fact let's go for that one’ [Ella, 22, 6–10, I].
Alarming imagery	‘yes um I look like a witch there (smoking) I look horrible I mean seeing yourself older isn't nice but that's definitely well (.) talk about beauty standards that one (non-smoking) is definitely more preferable’ [Mary, 25, 1–5, I].
Perceived Susceptibility	
Self-identification	‘the fact is when it got to 37 I looked young and round faced and youthful (non-smoking) but on the smoking one that's really where you start to see the difference (.) so that's why I kept on moving it back (.) I'm sure it's when I was 37 that was when it really kinda hit home and I was a bit like that's only in a couple of years’ [Ella, 22, 6–10, I].
	‘the whole image stuck with me for a bit I think cus it was actually me rather than some ladies hands or some bad smokers ugly lungs it was me and I think because it was a bit more personal like that I took it on a bit more seriously’ [Mary, 25, 1–5, FG].
Family resemblance	‘The left one [non-smoking image] looks like my grandma when she was 72 (.) my grandma was a non-smoker by the way urgh and the right one looks like my mum does now and she's only sixty she's a heavy smoker and all I can think is botox and filler can't sort that out’ [Laura, 39, 16–20, I].
	‘There was some disbelief with me I thought my mum that's eighty (.) she looks like that anyway and she hasn't smoked a cigarette’ [Elaine, 48, 11–15, FG].
Intention to Quit	
Intentions changed immediately	‘um yeah I'm planning (.) I would like to think I'm not going to smoke anymore (.) I'm going to quit…. just seeing that and I refuse to have a saggy chin and too many lines (.) I want to look good when I'm seventy-two’ [Ella, 22, 6–10, I].
Intentions changed for the future	‘quitting would be doable it wouldn't be horrible difficult or really boring (.) it would be um it would probably make me feel really good about myself so that was nice (1) so (.) its strengthened my intentions for the future and it's nice to think if I did quit I would get better skin for longer’ [Claire, 52, 16–20, I].
Intentions not changed	‘I reckon I will continue smoking for the foreseeable future just because like all of my friends all of my family everyone I know smokes so if I decide to quit it would just be so difficult you know what I mean?’ [Jody, 19, 11–15, I].

Note: Numbers in round brackets indicate the length of pauses within seconds e.g. (2), pauses less than a second are indicated by (.). Information in square brackets indicate- participants pseudonym, age, number of cigarettes smoked on a usual day and lastly source of quote (I = interview or FG = focus group).

### 3.1 Theme One

The theme ‘Health versus Appearance’ appeared throughout the transcripts and included three subthemes that explore both appearance and health concerns, and their combination (Table 2). Despite differences level of smoking behaviour, similarities were displayed in both appearance and health concerns expressed. Most participants expressed feeling more engaged with the appearance aspect of the intervention compared to more health-based approaches, though a minority did show a preference for health information (Table 2). A number of participants also stated that the appearance focus of the intervention supported visualizing how smoking would affect their internal physical wellbeing as they aged and supported existing goals and efforts to change their behaviour, solidifying their intentions to quit (Table 2). Results suggest that both health- and appearance-focused messages were important.

### 3.2 Theme Two

A major theme was ‘Shock Reaction’, which includes two subthemes: ‘Shock’ and ‘Disturbing imagery’. The theme captures accounts of shock from all women with varying reported smoking habits (Table 1, Table 2), indicating that ‘Shock Reaction’ was a consistent theme. Shock included feelings of shock, anxiety and fear. Mild shock was linked to an interest with the intervention content, while intense feelings of shock were expressed and linked to the smoking-aged images. Further to this, some participants used common negative archetypal images such as the “witch” to describe the aged image, juxtaposing this to the (more positive) non-smoking image (Table 2).

### 3.3 Theme Three

The theme ‘Perceived Susceptibility’ encapsulates how identifying with the smoking-aged images affected the participant experience. Two subthemes emerged, ‘Self-identification’, and ‘Family resemblance’ (Table 2). Some participants were able to relate to the aged image, mentioned the damaging effects of tobacco on their faces (or lack of), and expressed feelings of susceptibility to these effects in the future. Perceived susceptibility (to tobacco- damaging effects) was directly linked to the novelty of the intervention and seeing the aging effects on their own faces, as the threat felt personal. Participants indicated that the images were easier to relate to compared to traditional health-related stop-smoking images (e.g., images on cigarette packaging). Conversely, participants who did not self-identify with the images found difficulty in imagining the aging effects of smoking. If participants saw a resemblance between the age-morphed image and an older family member who either did or did not smoke, it enabled them to perceive the intervention as realistic. This roots the intervention images in realism, and demonstrates the individual differences in reactions of the women; if the resemblance with the family member was not consistent with that family member's smoking behaviour, the participant's belief in the validity of the images was reduced (Table 2).

### 3.4 Theme Four

‘Intention to Quit’ includes three subthemes key to its understanding: ‘Intentions changed immediately’, ‘Intentions changed for the future’ and ‘Intentions not changed’ (Table 2). The subtheme ‘Intentions changed immediately’ demonstrates that participants stated they would change their behaviour as a result of the intervention which was linked to a perceived susceptibility and motivation to change how their skin will age. Intentions to quit were demonstrated by those smoking fewer or more cigarettes, showing that the intervention has scope to impact a wide range of women (Table 2). Other participants did not express an immediate change in intentions, but did suggest that the intervention had strengthened their resolve to quit in the future, linked to appearance concerns. Finally, a small subgroup of participants, all with low levels of smoking behavior, stated that the intervention had not made any impact on their intentions (Table 2). This was likely due to the social norm of smoking outweighing concerns for both health and facial appearance at that time, cueing a continuation of smoking until their social worlds changed.

## 4 Discussion and conclusion

### 4.1 Discussion

Through qualitative analysis, four themes were identified that inform our understanding of 19–52 year old women's experiences of an age-progression intervention for smoking cessation; ‘Health versus Appearance’, ‘Shock Reaction’, ‘Perceived Susceptibility’ and ‘Intention to Quit’. The current study presents novel findings exploring women smokers' experiences of the intervention in a new era of facial aging technology where appearance-focused (embedded into apps such as Instagram) and health technologies are becoming increasingly common [18,20].

Importantly, ‘Health versus Appearance’ is a theme that has not been identified in previous research on age-progression interventions for smoking. The theme suggests a majority preference for appearance, or a combination of appearance and health-related goals. Although all bodies age, western societies have historically focused on, and problematized, ageing in women [8]. Accounts from women in the present study echo this focus, showing the age-appearance aspects were relevant to the participants. However, appearance was not the only motivation for smoking cessation, and many women focused on health as well as appearance, with some reporting that appearance-focus led to health concerns and motivation to quit while being interesting and engaging to view. This dual focus therefore might be related to a change in perspective regarding the value of wellness or health in women. Health focus, or being ‘health conscious’, has been projected into the mainstream and commands resources [39] and women specifically are growing up in digitized cultures where health promotion is evident on social media spaces [40]. Alongside this, appearance is commonly emphasized in social media, examples including built-in appearance-editing software in apps such as Instagram and Facebook [19] alongside the use of other common photo editing apps [18]. As both of these trends are set to continue a comprehensive approach, in the form of age-progression interventions and health/related advice, recognising both sets of goals could interest a wider range of women who smoke in a number of healthcare and patient settings, increasing chances of smoking cessation attempts. Previous research on age-progression interventions has observed some success with this kind of approach, as additional health-related stop smoking advice has been introduced at the end of the intervention to capitalize on motivation to quit [9]. Moreover, the appearance-focused intervention has been placed in traditional health-based settings such as pharmacies [9] or clinical waiting rooms [14] with some success. The use of this kind of intervention in these settings with women smokers, and with an equal focus on appearance and health, may be a positive way forward for work in this area. Therefore, the communication of health education in this way can be used as a tool to promote health in a range of clinical settings.

The theme ‘Shock Reaction’, found in previous work on age-progression interventions [33,16], was present within the current data. Participants appeared shocked by the smoking aged images, and were more positive about the non-smoking natural aged images as in previous research on UV exposure [17]. This focus on natural ageing does not necessarily perpetuate maintaining or reclaiming youthful appearance or promote an ageist orientation that often occurs in women. Instead, as explored above, intervention images accompanied with health information could be used to spotlight future consequences of harmful behaviours, such as smoking, alcohol consumption, or UV exposure. ‘Shock’ has been linked to increased arousal, [41] and to positive impacts on smoking cessation [42]. It is increasingly interesting that the theme of ‘Shock’ is a continued theme for age-progression interventions for smoking cessation (e.g. Flett et al., 2017; Grogan et al., 2010). Assumptions could be made that as appearance-based technology becomes more common in our society [18,19], that the “shocking” nature of this intervention type would diminish through means of desensitisation, as previously observed in the use of other smoking-based imagery [43]. However, in the current sample ‘Shock’ was still a dominant theme; this may have been due to the side by side presentation of the participant's smoking versus non smoking images and asking the participants if they could see any differences. Asking this specific question may have forced the participants to focus on the detrimental effects of smoking on their own skin and face. Communicating the intervention in this way may have reinforced the intervention message, as recommended for fear appeal messaging [44], and emphasised the personal relevance of the harmful effects of smoking. This has particular relevance for the current sample of women smokers. Women may experience increased positive effect from smoking [2], possibly explaining why it may take a more personalised approach to overcome the positive effect of smoking. Therefore, continuation of this delivery approach for interventions of this type may enhance delivery of health education, and specifically smoking cessation in women.

Personal relevance of the intervention was evidenced through the theme ‘Perceived Susceptibility’ linked to the theme ‘Intention to Quit’. Through viewing the intervention, perceived susceptibility to the harmful effects of cigarette smoke on facial wrinkling and in turn health could be increased. Protection Motivation Theory (PMT) suggests that high levels of perceived personal risk is a key component to behaviour change [45], therefore by showing the participant personal images related to smoking cessation intentions to quit could be increased, over that achieved by traditional health-based warning messages. Conversely, some of the participants did not perceive risk or identify with the aged intervention images, and a small subgroup of them did not express intentions to quit after viewing the intervention, with some expressing that smoking was a social norm within their existing relationships. In line with PMT [45] these participants may either not have perceived personal threat from damaging effects to the skin, or the threat elicited by the intervention may not outweigh the advantages of social interaction associated with their existing smoking behaviour. The Health Belief Model (HBM) may also add weight to this explanation as research indicates that perceived barriers are powerful in terms of behavioural outcomes [46], while both perceived threat and susceptibility remains strong predictors of smoking cessation [47], specifically influencing intentions to quit in women [48]. Therefore, these dimensions support how perceived susceptibility, emphasized by the personal nature of the intervention, can have a strong influence on intentions to quit smoking, while a barrier may be the perceived loss of social connections if smoking cessation was to occur. The HBM goes beyond PMT in terms of suggesting the additional component of self-efficacy moderates perception of threat in promoting behaviour change [49]. Future research could seek to measure and assess self-efficacy in relation to the intervention success.

One of the strengths of the study is the use of a rigorous qualitative approach, employing both individual interviews and confirmation of themes through focus groups with the same participants. In addition, the interdisciplinary team of authors engaged in reflexive analysis throughout the research process to ensure that preconceived judgments about the sample had as little impact on the analysis and interpretation of data as possible. The main limitation of the study was the ethnic homogeneity (white women predominantly), limiting the transferability of the present findings to other groups of women. Although appearance ideals for women are becoming increasingly homogeneous [50], it will be important for future work to investigate impacts of age-appearance facial morphing technology in a more diverse sample of participants. Furthermore, although women between 18 and 55 years were targeted for the study, the majority of participants were under 34 years; future research should recruit more women over 34 years, to enable investigation of differences in intervention perceptions in younger and older women. The intervention software utilised here is only normally available within health-related settings [34], so may not be accessible to some women smokers which may limit the potential of this approach. Future work might investigate whether the themes that emerged here are found when women are exposed to facial ageing on mobile apps such as have been used in previous studies [e.g. 21]. Lastly, focus groups were conducted between one and four weeks after the intervention/interview session as participants were recruited in blocks of five before focus groups were scheduled. This resulted in differing lengths of time between experiencing facial morphing and the focus groups. Although we limited the time difference to two weeks maximum, and used visualisation techniques to aid memory of the intervention experience, future research could aim to standardise time between morphing and focus groups.

Reflexivity was engaged in throughout the analysis process in order to present the women's accounts of the intervention fairly, although we recognize that our attitudes and intentions have impacted on the interpretation of these accounts. At the time the study took place, the first author (interviewer) was a PhD researcher in psychology. The second and third authors are also women and have experience in health psychology and investigating appearance-based interventions. The last author has extensive experience of stress effects on health and behaviour, the remaining authors have extensive experience researching behaviour change techniques. The analysis produced benefits from both experience with smoking cessation research and new interpretation of the subject. The method of data collection and analysis reflects the care taken by the researchers in handling a sensitive topic related to appearance, in the context of smoking behaviour.

### 4.2 Innovation

The study contributes to innovation in health care by adding to evidence regarding age-progression intervention approaches, through updating our understanding of the perceptions of the intervention within current society. The results demonstrate how the intervention approach could be effective in communicating risk of smoking to audiences (specifically women), where solely health information has previously been the main focus. The authors suggest innovation within the use of the intervention, including the recommendation it could be implemented in health-based settings such as doctor waiting rooms and pharmacies as previously trialled [9,14]. Implications and suggestions for innovation for practice in these settings include the following: i. Little training is needed to implement the approach. As this research is the first to provide scripted instructions for best practice practitioners could adhere to these instructions when delivering the intervention in order to maintain consistency in delivery. ii. The research emphasizes the importance of providing both appearance and health-based messages to maximize impact. As the intervention is recommended to be delivered within a health setting, the appearance-based nature of the intervention could be effectively combined with advice from health practitioners (e.g. pharmacists or stop smoking advisors) or health-based deliverables (e.g. leaflets and stop smoking advice websites) which the patient can use to reinforce initial action from viewing the aged images iii. The intervention, if implemented appropriately, has cost saving implications given the long-term health consequences of smoking. Future research can build on the information gained from the current study, by introducing a combination of the age-progression intervention with other intensive health-based approaches (such as nicotine replacement therapies or cognitive-behavioural therapies). Furthermore, future research could investigate short- and long-term smoking cessation outcomes in relation to the ‘Shock Reaction’ experienced during the intervention. Lastly, future research could continue to investigate the efficacy of these types of interventions in patient focused settings, applying app-based technology to facilitate public and patient engagement.

### 4.3 Conclusion

The current study provided novel findings regarding women smokers' attitudes to, and perceptions of, a facial-morphing age-progression intervention. Results indicate that despite growing prevalence of appearance-based apps, age-progression interventions for smoking cessation remain impactful and shocking to the viewer. Specifically, the results indicate that both appearance- and health messages could be incorporated together to optimize chances of smoking cessation; easily achievable given both interest and technological advancements in the area of e-health. In addition, the shock reaction, interlinked with personal relevance and perceived susceptibility, remains a strong theme which in turn is suggested to influence quit intentions.

## Funding

The primary author was supported by the Vice-chancellor scholarship at Manchester Metropolitan University. CJA is supported by the NIHR Manchester Biomedical Research Centre and the NIHR Greater Manchester Patient Safety Translational Research Centre.

## Declaration of Competing Interest

None to declare.

## Appendix A Supplementary data

Supplementary material Image 1
