# Supplementary material for: UK women smokers' experiences of an age-progression smoking cessation intervention: Thematic analysis of accounts
Source: PEC Innov. 2022 Feb 7;1:100021. doi: 10.1016/j.pecinn.2022.100021 (PMC10194392; doi:10.1016/j.pecinn.2022.100021)
Supplement: Supplementary file 1 — Supplementary material [file mmc1.docx]

**Supplemental Material 1. Interview Performa.**

**INTERVIEW PROFORMA**

Thank you for agreeing to take part in this interview.

As we told you when you agreed to take part, we are exploring procedures for a study investigating whether showing women the effects of smoking on how their faces age can help them to stop smoking.

The age-progression intervention that you have seen has shown you how your face is likely to age if you give up smoking compared to how it will age if you continue to smoke.

We want to know about how you felt about the intervention, the questionnaire, and all the procedures. Any comments on how to make it more effective will be useful to us. **No names will be recorded so that any information you provide will be anonymous.**

The Age-progression Intervention

1. So first of all, were you happy with the photograph taken before it was morphed?
2. On a scale of 1-5 how happy were you with the photo?
3. what did you think about the intervention?
4. How did you feel when you were doing it?
5. How did you feel immediately afterwards?
6. Did it affect your intention to smoke?
7. How could we make it more effective?
8. Were the instructions clear?

The Questionnaire

1. What did you think about the questionnaire?
2. Were there any parts of the questionnaire that made you uncomfortable? If so, what were they?
3. Were all sections clear? If not, which ones were not?
4. Are there any improvements you think we could make on this?

Physiological Measurement

1. How did you feel about the finger clips/electrodes?
2. Did you feel that having the electrodes on your fingers distracted you at all from focusing on the age-appearance images?
3. Is there anything we could have done to make this part of the study more comfortable for you?
4. Is there anything else that you would like to add?

Thank you for taking part…followed directly by **DEBRIEF**

**Supplemental Material 2. Intervention Instructions.**

I am now going to show you the intervention. (open laptop/tablet, open April or webcam).

I am going to take a picture of your face, please position yourself in the centre of the screen and keep a neutral expression, (for participants with glasses ask to remove).

(load picture in APRIL and fill in set up information) what is your age and ethnicity?

I am now going to edit your picture to match the stock image and match up the points of the face.

(once finished set up) On the screen you will see 2 pictures of your face, both pictures will age up to 72 each time, the one on the left will always be non-smoking and the one on the right will be with the effect of smoking.

*Morph2D* please can you close your eyes and open them when I tell you to. You will see your face aged to 72.

Open your eyes; can you see any differences between the images?

*Morph2D_R -* I am now going to show you the aging process, when I press play you will see both images age to 72. (Press play) can you see any differences?

*Morph2D_R’ -* I am going to repeat the aging process again now (press play) Can you see any other differences?

*Morph3D -*I am now going to change the pictures to a 3D image of your face, I am going to show you again the aging process and after you can move your own face around to view the sides and underneath. (give demonstration), (Press play) can you see any differences?

You can now use the mouse or keypad to move your face around (give demonstration).

*Morph3D_R -* I am going to repeat the aging again in the 3D view (press play) Can you see any more differences? Would you like to move your face around again?

*Participant Lead intervention time-* You can now have a look at the intervention on your own, you can drag the progress bar to different age levels. Notify me when you have finished
